# Assessment of Antimicrobial and Antioxidant Activities of *Nepeta trachonitica*: Analysis of Its Phenolic Compounds Using HPLC-MS/MS

**DOI:** 10.3390/scipharm85020024

**Published:** 2017-05-15

**Authors:** Ekrem Köksal, Hatice Tohma, Ömer Kılıç, Yusuf Alan, Abdülmelik Aras, İlhami Gülçin, Ercan Bursal

**Affiliations:** 1 Department of Chemistry, Faculty of Science, Erzincan University, Erzincan 24100, Turkey; koksalekrem@gmail.com (E.K.); htohma@erzincan.edu.tr (H.T.); 2 Department of Biology, Faculty of Science, Bingol University, Bingol 12000, Turkey; omerkilic77@gmail.com; 3 Department of Biology, Faculty of Science, Muş Alparslan University, Muş 49250, Turkey; y.alan@alparslan.edu.tr; 4 Department of Chemistry, Graduate School of Natural and Applied Sciences, Dicle University, Diyarbakır 21280, Turkey; abadi.49@hotmail.com; 5 Department of Chemistry, Faculty of Science, Atatürk University, Erzurum 25240, Turkey; 6 Department of Nursing, School of Health, Muş Alparslan University, Mus 49250, Turkey; ercanbursal@gmail.com

**Keywords:** *Nepeta trachonitica*, antimicrobial activity, antioxidant activity, phenolic compounds, HPLC-MS/MS

## Abstract

Continuing our work on the sources of natural bioactive compounds, we evaluated the antimicrobial and antioxidant activities of *Nepeta trachonitica* as well as its major phenolic content using the high-performance liquid chromatography-mass spectrometry/mass spectrometry (HPLC-MS/MS) technique. For antioxidant activity, ferric reducing antioxidant power (FRAP) and cupric ion reducing antioxidant capacity (CUPRAC) methods were performed to measure the reducing power and 1,1-diphenyl-2-picrylhydrazyl (DPPH) assay was employed to evaluate the radical scavenging activity of the sample. For antimicrobial activity, three Gram-positive and four Gram-negative microbial species as well as three fungi species were tested. *N. trachonitica* appeared to have reasonable antioxidant activity and decent antimicrobial activity as indicated by the inhibition of the organisms’ growth. The most susceptible species were *Bacillus subtilis* ATCC 6633 and *Escherichia coli* ATCC 11229 among the organisms tested. Ethanol extract of the plant has the highest effect on *Saccharomyces cerevisiae* but no effect on *Yarrowia lipolytica.* The HPLC-MS/MS analysis showed that at least 11 major phenolic compounds of *N. trachonitica* exist, the major ones being rosmarinic acid, chlorogenic acid and quinic acid. The obtained results suggest that *N. trachonitica* could be a promising source for food and nutraceutical industries because of its antimicrobial and antioxidant properties and phenolic compounds.

## 1. Introduction

Phenols are a main group of secondary metabolites synthesized in plants and provide colour and flavour for most fruits and vegetables [1,2]. Although phenols were considered as non-nutritive agents, they gain much more attention due to their many beneficial effects on human health [3]. For example, there are some epidemical and in vivo studies indicating that their consumption is associated with reduced cardiovascular diseases risk [4] or certain types of cancer [5]. Their mode of action could be due to their role as antioxidants, since they could stop or prevent the initiation of macromolecules oxidation in the cell [6]. Antioxidants can eliminate free radicals and reactive oxygen species (ROS), thus protecting the human body from their harmful effects, slowing the progression of many chronic diseases [7]. Therefore, due to the necessity to identify alternative natural antioxidant sources, the search for natural antioxidants originating from plants has considerably increased recently [8]. The other area in which antioxidants are commonly used is food and pharmaceutical industries to provide protection against oxidative degradation of foods [9,10]. Therefore, there is growing and serious interest in safer and natural originated antioxidants [11,12].

Besides having antioxidant properties, phenols may also act as antimicrobials. Antimicrobials are industrially important as they prevent foods from spoilage by microorganisms [13]. Although preserving foods using preservatives of chemical origin is possible, it is not preferable due to the adverse effect on human health. Therefore, natural and safer alternatives are demanded to extend the shelf life of foods [14,15]. Taken together, as bioactive compounds, naturally occurring phenols are important to preserve food and biomolecules from free radical- or microorganism-induced oxidation [16,17].

With multiple biological roles, the quantification and identification of phenols are important [18,19]. Different flavonoids and their derivatives could be specific in each plant sample [20,21]. The genus *Nepeta* is distributed in the southern and central regions of Europe, in the Middle East and Asia, comprising about 250 species [22]. Most of *Nepeta* taxa are Irano-Turanian elements and Turkey is one of the centres of diversity for the genus *Nepeta*. In Turkish flora *Nepeta* is represented by 40 taxa, 16 of which are endemic (ca. 40%) [23].

Throughout the world, *Nepata* species have been widely used in folk medicine due to its antitussive, diuretic, anti-asthmatic, antiseptic, antispasmodic, and febrifuge activities [24]. Aerial parts of *N. trachonitica* were used as tea and for haemorrhoids in the vicinity of Pertek in the Tunceli province of Turkey [25]. Also, it was reported its oils had anticandidal effect [26]. Additionally, we have not found any literature about the toxicity of *N. trachonitica*.

*N. trachonitica* is a perennial plant. It has several erect, sturdy and quadrangular stems, measuring 35–110 cm. It is unbranched, glabrous or finely pilose. Also, it has dark reddish-purple corolla, measuring ca. 20 mm. Its tube is narrow, straight, clearly exerted from or included in teeth of calyx. Generally, its habitat is rocky slopes, in *Quercus* scrub, at a height of 1100–2150 m [27].

For different *Nepeta* species, different biologically active compounds were determined. For example, essential oil of *Nepeta hindostana* was found to be effective on *Escherichia coli*, *Erwinia herbicola* and *Aspergillus ochraceu* as an antimicrobial source [28]. Moreover, essential oils of *Nepeta asterotricha* from Iran exhibited remarkable antimicrobial activity against various Gram-positive and Gram-negative bacteria [29]. Furthermore, it has been found that *Nepeta praetervuisa* leaves have antitumor activity with an 85% inhibition rate compared to standard drugs [30].

In the present study, we investigated the phenolic contents, antimicrobial and antioxidant activities of *N. trachonitica*; to our knowledge, *N. trachonitica* has not been investigated for these properties. The antioxidant properties of *N. trachonitica* were evaluated with three different antioxidant assays including cupric ion reducing antioxidant capacity (CUPRAC), ferric reducing antioxidant power (FRAP) and 1,1-diphenyl-2-picrylhydrazyl (DPPH) assays. Different microbial species were used for screening of antimicrobial activity of ethanolic plant extract. Finally, we performed high-performance liquid chromatography-mass spectrometry/mass spectrometry (HPLC-MS/MS) analysis of ethanol extraction of the sample.

## 2. Materials and Methods

### 2.1. Plant Samples

*N. trachonitica* was collected in Harput (Elazığ, Turkey); located south of Ölbe, on rocky slopes, 1300–1350 m in height, on 16 June 2014, collection number Kilic 5846. The plants were identified based on *The Flora of Turkey and East Aegean Islands, Volume 7* [27]. The voucher specimens are deposited at the herbarium of the Department of Biology, Hacettepe University Ankara and the Department of Park and Garden Plants of Bingol University, Turkey.

### 2.2. Preparation of the Extract

The sample powder was prepared by grounding 25 g air-dried *N. trachonitica* in a blander. For ethanol extract preparation, sample powder was mixed with absolute ethanol and stirred for one day at room temperature. The residue was re-extracted until extraction solvents became colourless (total solvent is 500 mL). The obtained extracts were filtered over Whatman paper (No. 1) and the filtrate was collected. Then, the ethanol in collected fractions was removed using a rotary evaporator (RE 100 Bibby, Stone Staffordshire, UK) at 30 °C. For water extract preparation, the same amount of sample powder (25 g) was mixed with 500 mL of distilled water on a magnetic stirrer for 24 h at room temperature, and lyophilized under 5 μm Hg pressure at −50 °C (Labconco, Freezone, Japan). Then, the samples placed in a tightly capped plastic bottle were kept at −20 °C until used for experiments [31].

### 2.3. Antioxidant Activity Studies

#### 2.3.1. CUPRAC Assay

For measurement of cupric ions’ (Cu^2+^) reducing capacities (CUPRAC assay) of *N. trachonitica* extracts, the method of Apak et al. [32] was performed as described previously [33]. Different sample extract concentrations (10–30 µg/mL) were added to a premixed reaction mixture containing 0.25 mL of CH_3_COONH_4_ buffer solution (1.0 M), 0.25 mL of ethanolic neocuproine solution (7.5 × 10^−3^ M), 0.25 mL of CuCl_2_ solution (0.01 M). After adjusting the final volumes to 2 mL with distilled water, absorbances were measured at 450 nm after 30 min incubation in room temperature. Increased absorbance was considered as increased reducing capacity [34].

#### 2.3.2. FRAP Assay

Fe^3+^ ion reducing power of the sample was evaluated according to the method of Oyaizu [35] and described previously [36]. The samples were prepared in distilled water at different concentrations and mixed with phosphate buffer (2.5 mL, 0.2 M, pH 6.6) and potassium ferricyanide [K_3_Fe(CN)_6_] (2.5 mL and 1%). The reaction mixture was incubated at 50 °C for 20 min. Then, 0.5 mL of FeCl_3_ (2.5 mL and 0.1%) and trichloroacetic acid (10%) were added to the reaction mixture, respectively. The increases in the absorbance were measured at 700 nm as an indication of reducing capacity.

#### 3.3.3. DPPH Assay

Radical scavenging abilities of the samples were evaluated using DPPH assay [37] as described previously [38]. Accordingly, samples at varying concentrations (10–30 µg/mL) were mixed with 750 µL purple coloured DPPH solution prepared in ethanol (1 mM). After the incubation of the mixture at room temperature for 30 min, radical scavenging activities of the samples were measured spectrophotometrically at 517 nm against blank samples, which contained alcohol. Decreased absorbance of the sample indicates DPPH free radical scavenging capability [39].

### 2.4. HPLC-MS/MS Analysis and Instrumentation

Prior to liquid chromatography-mass spectrometry/mass spectrometry (LC-MS/MS) analysis, 1000 mg extract/L was prepared by diluting dry filtrates with methanol and filtering through 0.2 µm microfiber filter. The LC-MS/MS analyses of the studied species were performed according to a previously validated method [40]. LC-MS/MS analyses of the phenolic compounds were performed by using an ultra-high performance liquid chromatography (UHPLC) coupled to a tandem MS instrument (Nexera model, Shimadzu, Kyoto, Japan). The liquid chromatograph was equipped with 54LC–30AD binary pumps (Shimadzu), a DGU-20A3R degasser (Shimadzu), a CTO-10ASvp column oven (Shimadzu) and a SIL-30AC autosampler (Shimadzu). The chromatographic separation was performed on a C18 reversed-phase Inertsil ODS-4 (150 × 4.6 mm, 3 µm, GL Sciences, Tokyo, Japan) analytical column. The sample was eluted using mobile phase A (0.1% formic acid, 5 mM ammonium formate and water) and mobile phase B (0.1% formic acid, 5 mM ammonium formate and methanol). The column temperature was fixed at 40 °C. The gradient program with the following proportions of solvent B was applied t (min), B%: (0, 40), (20, 90), (24, 90), (24, 40), (29, 40). The solvent flow rate and injection volume were settled as 0.5 mL/min and 4 µL, respectively.

MS detection was performed using a Shimadzu LCMS 8040 model triple quadrupole mass spectrometer equipped (Shimadzu) with an electrospray ionization (ESI) source operating in both positive and negative ionization modes. LC-MS/MS data were collected and processed by LabSolutions software (Shimadzu, Kyoto, Japan). To quantify the analytes, the multiple reaction monitoring (MRM) mode was used: the assay of investigated compounds was performed following two or three transitions per compound; the first one for quantitative purposes and the second and/or the third one for confirmation. The optimum ESI conditions were determined as desolvation line (DL) temperature; 250 °C, heat block temperature; 400 °C, nebulizing gas flow (nitrogen); 3 L/min and drying gas flow (nitrogen); 15 L/min.

### 2.5. Antimicrobial Activity

#### 2.5.1. Microorganisms

Test microorganisms included three different Gram-positive bacteria (*Bacillus subtilis* ATCC 6633, *Bacillus megaterium* DSM 32, *Staphylococcus aureus* ATCC 25923), four different Gram-negative bacteria (*Escherichia coli* ATCC 11229, *Enterobacter aerogenes* ATCC 13048, *Klebsiella pneumoniae* ATCC 13883, *Pseudomonas aeroginosa* ATCC 9027) and three fungi species (*Yarrowia lipolytica*, *Saccharomyces cerevisiae* and *Candida albicans* ATCC 10231)*.* Ampicillin/sulbactam (SAM-20), Rifampicin (RD-5), Erythromycin (E-15), Amikacin (AK-30) and Fluconazole (FCA-25) were used as positive controls.

#### 2.5.2. Microbiological Assay

The antimicrobial activity of ethanolic extract was determined by the disc diffusion method [41,42,43]. The ethanol solution containing 20 mg/mL of *N. trachonitica* extract (30, 60 and 90 μL) was absorbed on a 8 mm diameter sterile disc. A 1% rate of each microorganism from 10^6^ to 10^7^ CFU/mL suspensions was added to 15 mL sterile media (for bacteria Muller–Hintone agar, for yeast Sabourand 2% Glucose agar) to inoculate the media for assay. Each of these inoculated mediums was poured into Petri dishes (9.0 cm) and left at +4 °C for 1 h. Subsequently, discs prepared from *N. trachonitica* extract were added on these inoculated media and left again at +4 °C for 1 h. Four antibiotic standard discs were used as the positive controls. Sensitivity was deduced by comparing the inhibition zone diameter produced by the erythromycin (E-15 µg), ampicillin/sulbactam (SAM-20 µg), amikacin (AK-30 µg), rifampicin (RD-5 µg) and fluconazole (25 µg). The Petri dishes were incubated at 35 °C for 18–24 h, except for *C. albicans* ATCC 10231, *Y. lipolytica* and *S. cerevisiae* which were incubated at 27 °C. Inhibition zones were measured by a calibre and recorded as the mean diameter of three replications in mm [44,45].

## 3. Results and Discussion 

### 3.1. HPLC-MS/MS Analyses

In recent decades, research in nutrition and food science has been focused on plant products with potential biological activities including antioxidant and antimicrobial activities. Plant products are also rich in fibre, have no cholesterol and contain antioxidants such as flavonoids and others phenolic/polyphenolic compounds [44]. The yield of crude *N. trachonitica* extracts is found as approximately 15.02% and 10.12% for water and ethanol extracts, respectively. For identification and quantification of individual phenolic compounds found in *N. trachonitica* via HPLC-MS/MS analysis, we only used its ethanol extract. This is firstly because it was previously stated that ethanol has a protective role and prevents phenols from artificial oxidation by enzymes such as polyphenol oxidase. The second reason was that it is widely known that there is a correlation between phenolic content and antioxidant activity. So, we found that ethanol extract has better antioxidant activity; we only used ethanol extract for reasons of simplicity.

### 3.2. Identification of Phenols

**Figure 1:**
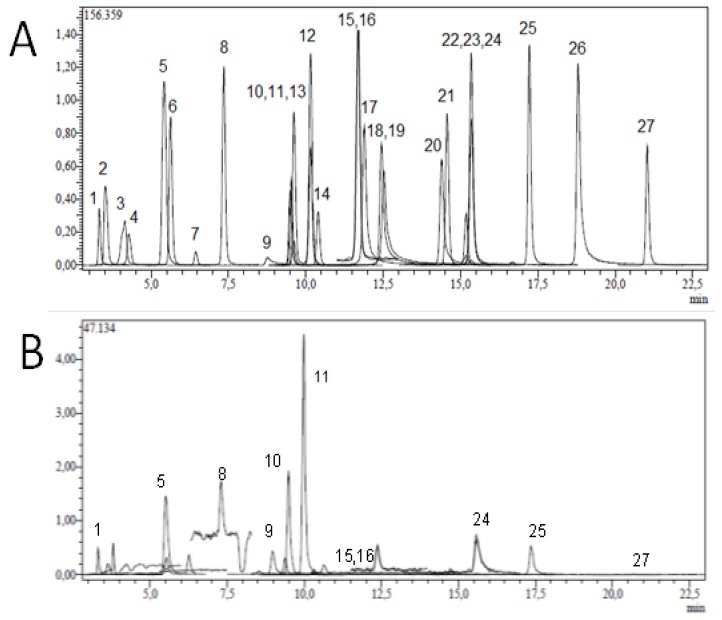
Typical HPLC chromatograms of (**A**) standards (**B**) of *Nepeta trachonitica* where (1) quinic acid, (5) chlorogenic acid, (8) *trans*-caffeic acid, (9) vanillin, (10) *p*-coumaric acid, (11) rosmarinic acid, (15) 4-OH benzoic acid, (16) salicylic acid, (24) kaempferol, and (25) apigenin (for all compounds see Table 2).

Figure 1 shows a typical HPLC chromatographic profile of the ethanol extract of *N. trachonitica* flowers. The identification of the peaks was made based on retention time, precursor ions and related fragment ions of the standards. The peaks identified are presented in Table 1. The most abundant phenolic compounds were determined as rosmarinic acid.

In the analysis of peak 1 by HPLC-MS/MS, a negative molecular ion at an *m/z* of 190.95 was identified as quinic acid. The peak 5 exhibited a negative molecular ion at [M − H]+ at *m/z* of 353 corresponding to chlorogenic acid. The peak 8 has shown an *m/z* of 178.95, indicating that this compound is *trans*-caffeic acid. The peak 9 exhibited an *m/z* of 151.05, which corresponds to vanillin. The peak 10 had an *m/z* of 162.95 corresponding to p-coumaric acid. The peak 11 has shown an *m/z* of 358.9, which is an indication of rosmarinic acid. The peak 15 exhibited an *m/z* at 136.95 corresponding to 4-OH benzoic acid. The peak 16 showed an *m/z* of 136.95. The corresponding compound was identified as salicylic acid. The peak 24 indicated an *m/z* of 284.95, which corresponds to kaempferol. Finally, the peak 25 exhibited an *m/z* of 268.95, which corresponds to apigenin. 

### 3.3. Quantification of Phenols

To quantify each phenolic compound in the sample, the individual peak areas of each component from the HPLC chromatogram profile were compared with the areas of standards at known concentrations. The amount of each compound was expressed as µg analyte/kg dried *N. trachonitica* extract. Because of the natural variation of the plants, there are differences in the amount of phenolic content specific to each plant and even to each plant part analysed. For example, total phenol and flavonoid content was variable in three *Celosia* species [46]. Furthermore, reversed-phase high-performance liquid chromatography (RP-HPLC) analysis of French Cider Apple Variety showed that the amounts of procyanidins were 892 mg/kg of fresh apple and 229 mg/kg of fresh apple in the epidermis zone and core zone, respectively.

In this study, the most abundant phenolic compound was rosmarinic acid (250.06 ± 12.25 µg/kg extract). The other plants rich in rosmarinic acid are *Thymus sipyleus* Boiss. [47], fennel [48], *Perilla frutescens* Mill. [49] as well as Lamiaceae taxa [50]. There are reports indicating that rosmarinic acid suppresses the glycolytic ATP production under aerobic conditions which is known as the Warburg effect, a universal property of most cancer cells [51]. The authors suggest that since there is a link between inflammation and tumorigenesis, the effect of rosmarinic acid on gastric carcinoma could be related to the inflammatory pathway as it inhibited proinflammatory cytokines [51]. The second and third most abundant compounds were chlorogenic acid (160.15 ± 7.84 µg/kg extract) and quinic acid (109.2 ± 5.2 µg/kg extract) (Table 1). These compounds were found to be high in other plants such as *Carica papaya* [52] and *Veronica* species [53].

### 3.4. Antioxidant Activity

An antioxidant molecule is defined as a chemical that, even at low concentrations, delays or prevents the oxidation of substrate-like biomolecules [54,55]. Antioxidants are needed in industry to preserve the foods from oxidation and decay and more importantly are needed in the human body to preserve the biomacromolecules from oxidation. Therefore, evaluation of food and plants in terms of antioxidant capacity is of interest [56].

The selection and the number of methods to measure the antioxidant capacity is important, since there is no one ideal standard way of evaluating the antioxidant value of the food. One of the ways to measure antioxidant capacity of a product is to measure its radical scavenging activity, since free radicals occurring as a consequence of a normal physiology are capable of oxidizing cellular components [57]. The other way to evaluate the antioxidant activity of a product is to measure metal chelating activity, since metals such as Fe and Cu could possibly lead to the formation of OH radicals via Fenton reaction, which leads again to the oxidation of cellular components [58]. In the study, we examined the antioxidant property of *N. trachonitica* using three different assays. FRAP and CUPRAC assays were performed to evaluate the reducing antioxidant activity, whereas a DPPH assay was conducted to investigate the radical scavenging activity of the sample [59].

**Figure 2:**
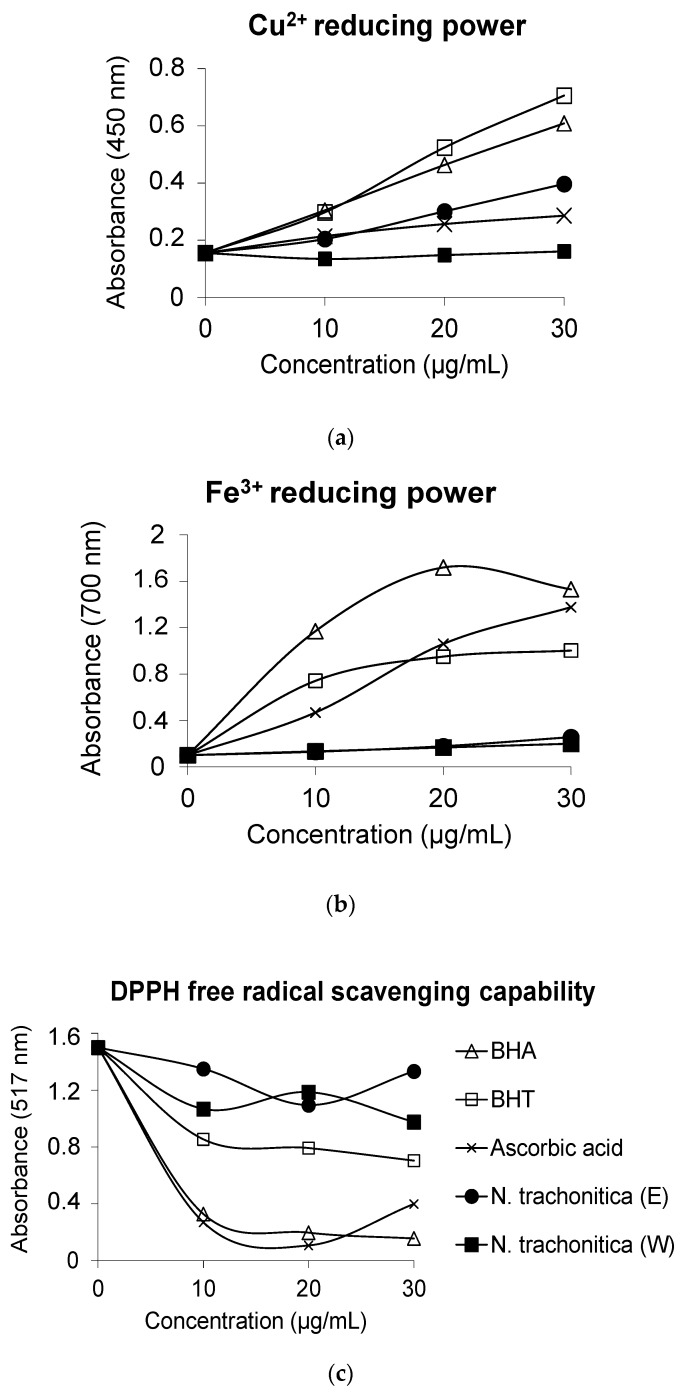
Antioxidant activity of *N. trachonitica* and standards by using (**a**) CUPRAC assay, (**b**) FRAP assay and (**c**) DPPH assay (E: ethanol extract of *N. trachonitica*, W: water extract *N. trachonitica*). BHA: Butylated hydroxyanisole; BHT: Butylated hydroxytoluene.

The result showed that in the CUPRAC assay, ethanolic extract of *N. trachonitica* has stronger reducing power activity than ascorbic acid, which is one of the most commonly used synthetic standard antioxidants (Figure 2a). However, water extract of *N. trachonitica* had the lowest scavenging activity in this assay. It was clear that increasing the sample concentration after 20 µg/mL did not further increase the cupric ions’ reducing capacity. In addition, in the FRAP assay, both ethanolic and water extract of *N. trachonitica* showed lower ferric ions reducing activity than ascorbic acid (Figure 2b). DPPH radical scavenging activities of *N. trachonitica* from both extracts were lower than all standard antioxidants used (Figure 2c).

These results are consistent with the previous studies. For example, it has been shown that the methanol extract and essential oil of *Nepeta cataria* L. had slight and no effect on DPPH radical scavenging activity [60]. However, there is also a report indicating that acetone extract of *Nepeta meyeri* Benth. was effective to scavenge DPPH radical. This could be due to the different solvent used in the extraction process or the usage of sample extract at high concentration (50 µg/mL) [61].

In this study, it was not surprising to see different antioxidant activity of the sample due to application of different antioxidant assays. Previously, it has been shown that the same plant could exhibit different radical scavenging capacity based on the assay performed. For example, a comparative study has shown that pine (*Pinus maritima*) extract has a 94% and 76% inhibition rate for DPPH and 2,2'-azino-bis(3-ethylbenzothiazoline-6-sulphonic acid) (ABTS), respectively [62].

In addition, the differences in antioxidant capacity of samples prepared in different solutions were also expected since the solvent used for extraction could affect the antioxidant capacity of the sample tested. For instance, the water and 50% methanol exactions of walnut green husk from Mellanaise cultivar exhibited 0.72 and 0.33 DPPH radical scavenging activity, respectively [63].

### 3.5. Antimicrobial Activity

The need for a substance with antimicrobial activity is increasing due to the alarming increase in infectious diseases across the world [64]. Plants produce these special substances that can have an effect on pathogens, either inhibiting their growth or killing them with little or no toxicity to the host [65]. It is widely known that phenolic compounds of plant origin may have antibacterial properties [66]. Although one pure phenolic component alone could be efficient enough to show antimicrobial activity, in this study, we preferred to use the total plant extract for antimicrobial activity as the property and behaviour of a bioactive component may change in the presence of other components due to a synergistic effect [67].

Antimicrobial activity of *N. trachonitica* was investigated against three Gram-positive (*B. subtilis*, *S. aureus*, and *B. megaterium*) and four different Gram-negative (*E. aerogenes*, *E. coli*, *K. pneumonia*, and *P. aeruginosa*) bacteria. We also tested whether the sample is effective against three fungus species (*Y. lipolytica*, *C. albicans*, and *S. cereviciae*). To determine the inhibition of bacteria growth, the inhibition zones were calculated for each sample concentration (Table 2). To evaluate the value of the sample, the reference antibiotics were also analysed for comparison.

The ethanolic extract of *N. trachonitica* has shown a strong and similar effect on *B. subtilis* and *E. coli* with the inhibition zone of 12.0 ± 1.24. The susceptibilities of both bacteria were dose dependent. Additionally, the highest antimicrobial activity against *B. megaterium* was observed when 90 µL sample extract was used. *E. aerogenes* was also sensitive to the sample extract and the effect was increased with increased sample amount. However, the sample extract exhibited a weak effect on *P. aeroginosa* and no effect on *K. pneumoniae*.

*N. trachonitica* also exhibited antifungal activity. *S. cereviciae* was the most susceptible to ethanolic extract of the sample, followed by *C. albicans.* The inhibition zones for these two fungi were 19.0 ± 1.69 and 13.0, respectively with a dose dependent manner. On the other hand, the sample showed no antifungal activity against *Y. lipolytica*. Overall, these data suggest that *N. trachonitica* has similar antimicrobial affects against certain bacteria as compared to standard antibiotics, but to a lesser extent.

## 4. Conclusions

In this study, antioxidant capacity, antimicrobial activity and phenolic composition of *N. trachonitica* were presented for the first time. Antioxidant and antimicrobial activity as well as phenolic composition of *N. trachonitica* were presented for the first time in this study. Antioxidant assays indicated that the plant extract has variable and reasonable antioxidant activity depending on the solvent and the methods used. Antimicrobial activity studies showed that the ethanolic extract of the sample has comparable results to antibiotic standards. Furthermore, with a powerful analytical HPLC technique, identification and quantification of 11 phenolic compounds was achieved. We identified the phenols based on an analysis of their mass spectrum and other relevant bibliographic information. In this context, the data presented in this work suggest that *N. trachonitica* could be a new source for phenols and antimicrobials, which is a challenging issue for manufacturers and food scientists. Further work might be needed to elucidate how the bioactive compounds of *N. trachonitica* inhibit the growth of microorganisms.

## Figures and Tables

**Table 1:** The phenolic acid composition of *Nepeta trachonitica* (µg analyte/kg extract).

No	Analytes	RT ^a^	Parent ion (*m/z*) ^b^	Ionization Mode	r^2 c^	RSD (%) ^d^	Linearity Range (µg/L)	LOD/LOQ (µg/L) ^e^	Recovery (%)	U ^f^	*Nepeta trachonitica*
1	Quinic acid	3.32	190.95	Negative	0.9927	0.0388	250–10,000	22.3/74.5	103.3	4.8	109.2 ± 5.2
2	Malic acid	3.54	133.05	Negative	0.9975	0.1214	250–10,000	19.2/64.1	101.4	5.3	ND
3	*trans*-Aconitic acid	4.13	172.85	Negative	0.9933	0.3908	250–10,000	15.6/51.9	102.8	4.9	ND
4	Gallic acid	4.29	169.05	Negative	0.9901	0.4734	25–1000	4.8/15.9	102.3	5.1	ND
5	Chlorogenic acid	5.43	353	Negative	0.9932	0.1882	250–10,000	7.3/24.3	99.7	4.9	160.15 ± 7.84
6	Protocatechuic acid	5.63	152.95	Negative	0.9991	0.5958	100–4000	25.8/85.9	100.2	5.1	ND
7	Tannic acid	6.46	182.95	Negative	0.9955	0.9075	100–4000	10.2/34.2	97.8	5.1	ND
8	*trans*- Caffeic acid	7.37	178.95	Negative	0.9942	1.0080	25–1000	4.4/14.7	98.6	5.2	28.97 ± 1.5
9	Vanillin	8.77	151.05	Negative	0.9995	0.4094	250–10,000	10.1/33.7	99.2	4.9	62.78 ± 3.08
10	*p*-Coumaric acid	9.53	162.95	Negative	0.9909	1.1358	100–4000	15.2/50.8	98.4	5.1	49.23 ± 2.51
11	Rosmarinic acid	9.57	358.9	Negative	0.9992	0.5220	250–10,000	10.4/34.8	101.7	4.9	250.06 ± 12.25
12	Rutin	10.18	609.1	Negative	0.9971	0.8146	250–10,000	17.0/56.6	102.2	5.0	ND
13	Hesperidin	9.69	611.1	Positive	0.9973	0.1363	250–10,000	21.6/71.9	100.2	4.9	ND
14	Hyperoside	10.43	463.1	Negative	0.9549	0.2135	100–4000	12.4/41.4	98.5	4.9	ND
15	4-OH Benzoic acid	11.72	136.95	Negative	0.9925	1.4013	25–1000	3.0/10.0	106.2	5.2	4.4 ± 0.23
16	Salicylic acid	11.72	136.95	Negative	0.9904	0.6619	25–1000	4.0/13.3	106.2	5.0	4.39 ± 0.22
17	Myricetin	11.94	317	Negative	0.9991	2.8247	100–4000	9.9/32.9	106.0	5.9	ND
18	Fisetin	12.61	284.95	Negative	0.9988	2.4262	100–4000	10.7/35.6	96.9	5.5	ND
19	Coumarin	12.52	146.95	Positive	0.9924	0.4203	100–4000	9.1/30.4	104.4	4.9	ND
20	Quercetin	14.48	300.9	Negative	0.9995	4.3149	25–1000	2.0/6.8	98.9	7.1	ND
21	Naringenin	14.66	270.95	Negative	0.9956	2.0200	25–1000	2.6/8.8	97.0	5.5	ND
22	Hesperetin	15.29	300.95	Negative	0.9961	1.0164	25–1000	3.3/11.0	102.4	5.3	ND
23	Luteolin	15.43	284.95	Negative	0.9992	3.9487	25–1000	5.8/19.4	105.4	6.9	ND
24	Kaempferol	15.43	284.95	Negative	0.9917	0.5885	25–1000	2.0/6.6	99.1	5.2	18.01 ± 0.92
25	Apigenin	17.31	268.95	Negative	0.9954	0.6782	25–1000	0.1/0.3	98.9	5.3	8.13 ± 0.43
26	Rhamnetin	18.94	314.95	Negative	0.9994	2.5678	25–1000	0.2/0.7	100.8	6.1	ND
27	Chrysin	21.18	253	Negative	0.9965	1.5530	25–1000	0.05/0.17	102.2	5.3	0.14

^a^ RT: retention time.; ^b^ Parent ion (*m/z*): Molecular ions of the standard compounds (mass to charge ratio); ^c^ r^2^: coefficient of determination; ^d^ RSD: relative standard deviation; ^e^ LOD/LOQ (µg/L): limit of detection/limit of quantification; ^f^ U (%): percent relative uncertainty at 95% confidence level (k:2); ND: not determined.

**Table 2:** Antimicrobial and antifungal activity results of *N. trachonitica.*

Microorganisms		Inhibition Zone Diameter (mm)							
		*N. trachonitica* (20 mg/mL Ethanol)			Antibiotics				
		30 µL	60 µL	90 µL	Erythromycin (15 µg)	Ampicillin/ Sulbactam (20 µg)	Amikacin (30 µg)	Rifampicin (5 µg)	Fluconazole (25 µg)
Gram positive	*B. subtilis*	9 ± 0.00	10 ± 0.81	12 ± 1.24	20 ± 1.24	14 ± 0.47	11 ± 1.24	21 ± 1.24	-
	*S. aureus*	-	-	-	21 ± 0.00	10 ± 0.81	9 ± 0.00	18 ± 1.69	-
	*B. megaterium*	10 ± 0.00	10 ± 0.00	11 ± 0.00	25 ± 1.69	-	10 ± 0.81	16 ± 1.24	-
Gram negative	*E. aerogenes*	9 ± 0.00	10 ± 0.47	11 ± 0.81	27 ± 1.24	10 ± 0.47	9 ± 0.00	16 ± 0.47	-
	*E. coli*	-	10 ± 0.47	12 ± 1.24	19 ± 0.00	13 ± 1.24	13 ± 0.81	18 ± 1.24	-
	*P. aeroginosa*	-	-	9 ± 0.00	19 ± 1.69	-	14 ± 0.00	8 ± 0.00	-
	*K. pneumoniae*	-	-	-	19 ± 0.47	16 ± 1.69	10 ± 0.47	19 ± 1.69	-
Fungus	*Y. lipolytica*	-	-	-	-	-	-	-	21 ± 0.00
	*C. albicans*	11 ± 0.81	13 ± 1.24	13 ± 0.00	-	-	-	-	23 ± 0.47
	*S. cereviciae*	12 ± 0.47	15 ± 0.47	19 ± 1.69	-	-	-	-	-
